# Morbid Dental Specimens

**Published:** 1845-03

**Authors:** 


*Morbid Dental Specimens.*—We have recently received, from Dr. A. Vancamp, of Nashville, Tennessee, a box containing nearly a hundred of interesting anomalous and morbid specimens of dental anatomy, and for which, we return him our warmest thanks. In the collection there are some singular examples of exostosis, and one of osseous union of the teeth; others exhibiting the pernicious effects of badly performed dental operations, and of the immoderate and imprudent use of mercury. Among the last, is nearly the half of the inferior maxillary of a child seven years old.—*Balt. Ed.*

